# Metformin exposure in first trimester of pregnancy and risk of all or specific congenital anomalies: exploratory case-control study

**DOI:** 10.1136/bmj.k2477

**Published:** 2018-06-25

**Authors:** Joanne E Given, Maria Loane, Ester Garne, Marie-Claude Addor, Marian Bakker, Bénédicte Bertaut-Nativel, Miriam Gatt, Kari Klungsoyr, Nathalie Lelong, Margery Morgan, Amanda J Neville, Anna Pierini, Anke Rissmann, Helen Dolk

**Affiliations:** 1 Administrative Data Research Centre Northern Ireland, Ulster University, Belfast BT37 0QB, UK; 2 Institute of Nursing and Health Research, Ulster University, Belfast BT37 0QB, UK; 3 Paediatric Department, Hospital Lillebaelt, Kolding, DK-6000, Denmark; 4 Medical Genetics, CHUV, Lausanne, CH-1011, Switzerland; 5 University of Groningen, University Medical Center Groningen, Department of Genetics, Eurocat Northern Netherlands, 9700RB, Netherlands; 6 Registre des Malformations Congenitales de la Reunion, Saint-Pierre, BP350, Ile de la Reunion; 7 Directorate for Health Information and Research, Guardamangia, PTA 1313, Malta; 8 Division of Mental and Physical Health, Norwegian Institute of Public Health, Bergen, Norway; 9 Department of Global Public Health and Primary Care, University of Bergen, N-5018, Norway; 10 Inserm UMR 1153, Obstetrical, Perinatal and Pediatric Epidemiology Research Team (Epopé), Center for Epidemiology and Statistics Sorbonne Paris Cité and DHU Risks in pregnancy, Paris Descartes University, Paris, 75014, France; 11 Congenital Anomaly Register and Information Service for Wales, Public Health Wales, Swansea SA2 8QA, UK; 12 IMER Registry (Emilia Romagna Registry of Birth Defects), University of Ferrara and Azienda Ospedaliero Universitaria di Ferrara, Ferrara, 44100, Italy; 13 Tuscany Registry of Congenital Defects, Institute of Clinical Physiology, National Research Council/Fondazione Toscana Gabriele Monasterio, Pisa, 56126, Italy; 14 Malformation Monitoring Centre Saxony-Anhalt, Medical Faculty Otto-von-Guericke University Magdeburg, Magdeburg, D-39120, Germany; 15 Institute of Nursing and Health Research, Ulster University, Belfast BT37 0QB, UK

## Abstract

**Objective:**

To investigate whether exposure to metformin during the first trimester of pregnancy, for diabetes or other indications, increases the risk of all or specific congenital anomalies.

**Design:**

Population based exploratory case-control study using malformed controls. Cases of 29 specific subgroups of non-genetic anomalies, and all non-genetic anomalies combined, were compared with controls (all other non-genetic anomalies or genetic syndromes).

**Setting:**

11 EUROmediCAT European congenital anomaly registries surveying 1 892 482 births in Europe between 2006 and 2013.

**Participants:**

50 167 babies affected by congenital anomaly (41 242 non-genetic and 8925 genetic) including live births, fetal deaths from 20 weeks’ gestation, and terminations of pregnancy for fetal anomaly.

**Main outcome measure:**

Odds ratios adjusted for maternal age, registry, multiple birth, and maternal diabetes status.

**Results:**

168 babies affected by congenital anomaly (141 non-genetic and 27 genetic) were exposed to metformin, 3.3 per 1000 births. No evidence was found for a higher proportion of exposure to metformin during the first trimester among babies with all non-genetic anomalies combined compared with genetic controls (adjusted odds ratio 0.84, 95% confidence interval 0.55 to 1.30). The only significant result was for pulmonary valve atresia (adjusted odds ratio 3.54, 1.05 to 12.00, compared with non-genetic controls; 2.86, 0.79 to 10.30, compared with genetic controls).

**Conclusions:**

No evidence was found for an increased risk of all non-genetic congenital anomalies combined following exposure to metformin during the first trimester, and the one significant association was no more than would be expected by chance. Further surveillance is needed to increase sample size and follow up the cardiac signal, but these findings are reassuring given the increasing use of metformin in pregnancy.

## Introduction

Metformin is an oral blood glucose lowering drug that has been used in the treatment of type 2 diabetes since the 1950s.^1^ Despite reservations about its use in pregnancy, metformin has been recommended for use in pregnancy in the UK since 2008 in women with gestational diabetes and in type 2 diabetes when the likely benefits outweigh the potential for harm.^2^ ^3^ The emergence of type 2 diabetes in children and younger women of childbearing age has driven a sharp increase in the prevalence of type 2 diabetes in pregnancy,^4^ ^5^ which, together with these recommendations,^3^ is likely to further increase the number of women exposed to metformin in pregnancy.

Metformin is also prescribed in polycystic ovary syndrome, in which it improves insulin sensitivity, may aid weight reduction, and helps to normalise the menstrual cycle (increasing the rate of spontaneous ovulation).^6^ Exposure to metformin in early pregnancy among women undergoing treatment for polycystic ovary syndrome may therefore occur. The use of metformin to prevent diabetes in pre-diabetic populations, as a cancer treatment,^7^ ^8^ and as a weight loss medication for non-diabetic obesity is also of interest.^9^ ^10^ Expansion of indications for metformin use will increase the risk of unintentional exposures during pregnancy.

The use of metformin in pregnancy remains controversial.^11^ ^12^ Metformin affects stem cell function and has been shown to cross the human placenta at term, exposing the fetus to concentrations approaching those in the maternal circulation.^13^ ^14^ ^15^ ^16^ ^17^ Animal studies have shown no increased risk of congenital anomaly at therapeutic doses,^18^ ^19^ ^20^ but these are limited in terms of predicting risk in humans.^21^ Three meta-analyses have been conducted to explore the risk of congenital anomaly following exposure to metformin in humans and have concluded that no evidence exists to suggest a significantly increased risk of all major congenital anomalies compared with maternal disease matched control groups.^22^ ^23^ ^24^ These meta-analyses were, however, based on small heterogeneous samples from studies that were not specifically designed to evaluate the rate of congenital anomalies.^22^ ^25^ A recently published cohort study based on 392 women exposed to metformin who contacted teratogen information services found an increased risk of major birth defects among women taking metformin for diabetes but not for other indications. The authors concluded that the increased risk was due to the underlying diabetes, but they did not have a diabetic comparison group.^26^ Any investigation of the risk of congenital anomaly associated with metformin is complicated by the fact that pregestational diabetes increases the risk of major congenital anomaly two to threefold.^27^ ^28^ Metformin, if used for diabetes or pre-diabetes, may lead to a decreased risk of congenital anomaly as a result of achieving better glycaemic control,^29^ to an increased risk of congenital anomaly due to independent teratogenic action, or both.

To date, the size of the exposed population covered in the literature is too small to rule out risks of specific malformations. EUROmediCAT, a population based reproductive pharmacovigilance system based on the European Surveillance of Congenital Anomalies (EUROCAT) network,^30^ ^31^ provides an opportunity to contribute much needed epidemiological evidence from a large population to the available literature. The aim of this study was to investigate whether exposure to metformin during the first trimester increases the risk of all or specific congenital anomalies.

## Methods

### Study design

We did an exploratory case-control study using malformed controls, using the EUROmediCAT central database. The use of malformed controls was initially proposed for birth defect epidemiology as a method of controlling for maternal recall bias (box 1).^36^ ^38^ It is used in EUROmediCAT to control for the source of drug exposure data and because data on non-malformed controls are not available.^39^

Box 1 **Case-control studies using malformed controls**

In a case-control study, a group of patients who have the disease of interest (cases) and a group who do not have the disease (controls) are selected, and the proportion with the exposure of interest in each group is compared.^32^

In a case-control study using malformed controls, commonly used for congenital anomaly studies,^33^ a group of babies with a particular congenital anomaly of interest (cases) and a group who have different congenital anomalies (controls) are selected. This design is particularly suitable for investigating the specificity of association between specific malformations and specific exposures, rather than the overall risk of malformation.^34^

Case-control studies using malformed controls were initially proposed to overcome maternal “recall bias.”^35^ Although this is not an problem for EUROmediCAT drug exposure data, which are mainly collected from prospective medical/maternity records, it is also a useful design when comparable data on non-malformed controls are not available.

The main potential disadvantage of using malformed controls is “teratogen non-specificity bias.”^36^ ^37^ This is where a teratogen causes many different malformations, some of which are included in the control group leading to an underestimation of risk. This can be avoided by excluding from the controls any malformation previously associated in the literature with the exposure in question and including a wide range of malformations in the control group, as no known teratogen increases the risk of all malformations to the same extent.^36^ An additional approach is to specify a control group of genetic syndromes, which cannot have been caused by environmental teratogens, but as their numbers are small this reduces statistical power.

### Study population and data

EUROCAT population based registries record all major congenital anomalies among live births, fetal deaths at 20 weeks’ gestation or later, and terminations of pregnancy for fetal anomaly, using ICD-10 (international classification of diseases, 10th revision) codes.^40^ ^41^ Detailed descriptions of registries and the methods used have been published previously.^30^ ^42^ ^43^ The EUROmediCAT database includes data, since 1995, from those EUROCAT registries that record first trimester drug exposure either directly or through linkage with healthcare databases with information on prescribing and dispensing of drugs.^44^ Exposure to metformin in the first trimester was rare before 2006, so this study was based on data from 2006 onwards. Registries with less than three exposures were excluded.

Congenital anomalies are classified to 91 standard “EUROCAT subgroups.”^40^ ^41^ These include hierarchical subgroups—for example, spina bifida is a subgroup that forms part of the subgroup “neural tube defects,” which forms part of the group “nervous system.” These subgroups are furthermore divided into non-genetic and genetic categories, according to whether a genetic syndrome has been diagnosed in association with the congenital anomaly subgroup in question.

### Case and control groups

Cases and controls were live births, fetal deaths from 20 weeks, and terminations of pregnancy for fetal anomaly.^41^ The literature contains no signals for specific congenital anomalies potentially associated with metformin that would suggest previous hypotheses to test as “cases.” We did an exploratory analysis in which, for each analysis, we considered a single non-genetic EUROCAT subgroup of congenital anomaly to be the “case” group.^40^ ^41^

We used two control groups. “Non-genetic controls” were the remaining babies with non-genetic congenital anomalies after exclusion of the specific congenital anomaly being analysed as the case group and of any subgroup at a hierarchical level above. Genetic controls included chromosomal anomalies, skeletal dysplasias, congenital skin disorders, genetic syndromes, and microdeletions.

When analysing hypospadias as a case group, we used only male controls. We excluded babies with isolated congenital hip dislocation/dysplasia owing to the association with large babies and potential for confounding.^45^ We excluded cases and controls with maternal epilepsy or exposure to antiepileptic drugs owing to the association with congenital anomalies.^46^ ^47^ We cleaned the limb reduction defect subgroup by searching anomaly text descriptions for shortening of the limbs. We then reviewed the diagnosis for cases with this text description and reclassified those that had been misclassified as limb reduction defects (31/122 (25%) cases with this text description, excluding Norway and Paris for which no text was available).

When interpreting the results, we divided case subgroups according to whether or not they had previously been associated with pregestational diabetes in the EUROCAT database, according to Garne et al^48^—that is, neural tube defects, congenital heart defects, omphalocele, and syndactyly. This identified which results were most at risk of confounding by indication, requiring a more cautious interpretation.

### Exposure

**Fig 1:**
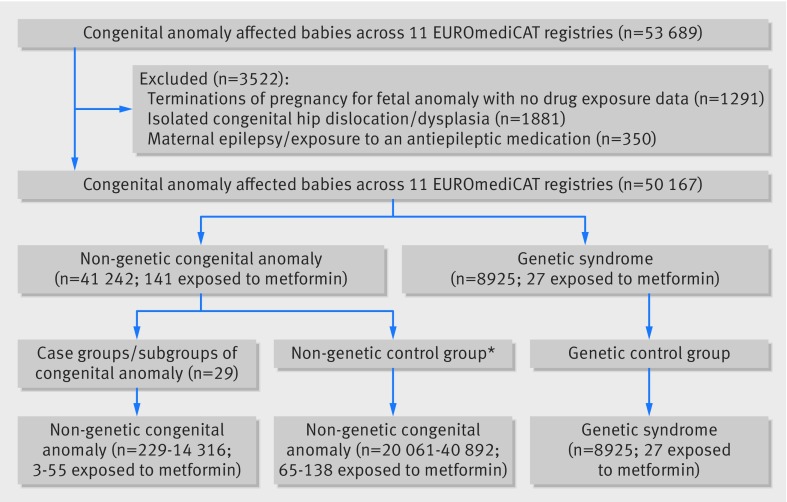
Flow diagram detailing number of congenital anomaly affected babies included in analysis. *Varies with case genetic anomaly; consists of all remaining non-case, non-genetic congenital anomalies excluding any congenital anomaly group at hierarchical level above case group

We obtained most data on maternal drug exposures in the first trimester retrospectively from prospective maternity records. Additional data sources available for some registries included the medical records of the infant, records from the general practitioner, pregnancy passports, and maternal interviews before or after birth.^31^ ^42^ ^49^ For the Norway registry, drug exposures were based on prescription redemption records during the first trimester of pregnancy. The supplementary table gives more details. We excluded all terminations of pregnancy for fetal anomaly in Emilia Romagna, as they had no information on drug exposure (fig 1). We recorded all drug exposures in the first trimester by using the World Health Organization’s Anatomical Therapeutic Chemical classification system.^50^ This is a hierarchical system, which allocates to a drug a code based on the organ or system on which it acts (first level) and its therapeutic (second level), pharmacological (third level), and chemical properties (fourth and fifth level). We defined exposure to metformin as use of metformin, or a combination product (two or more drugs in a single tablet) containing metformin, during the first trimester. The Anatomical Therapeutic Chemical codes used to identify metformin exposure were A10BA02 (metformin), A10BD02 (metformin and sulfonylureas), A10BD03 (metformin and rosiglitazone), A10BD05 (metformin and pioglitazone), A10BD07 (metformin and sitagliptin), A10BD08 (metformin and vildagliptin), A10BD10 (metformin and saxagliptin), A10BD11 (metformin and linagliptin), A10BD13 (metformin and alogliptin), A10BD14 (metformin and repaglinide), A10BD15 (metformin and dapagliflozin), A10BD16 (metformin and canagliflozin), A10BD17 (metformin and acarbose), A10BD18 (metformin and gemigliptin), and A10BD20 (metformin and empagliflozin). For all registries, we defined the first trimester as the period from the first day of the last menstrual period to the end of gestational week 12.

Data on maternal illness before and during the first 20 weeks of pregnancy were recorded, mostly prospectively from maternity records, using ICD-10 codes.^41^ This provided information on the potential indication for metformin use. In Norway, indications other than maternal diabetes were not recorded. We therefore excluded the Norway registry from all analyses relating to polycystic ovary syndrome and infertility. Registries individually verified all metformin exposures (cases or controls), the first trimester timing, indication for prescribing, and the malformation.

### Statistical analysis

#### Descriptive analyses of metformin use and risk of congenital anomaly associated with disease indications

We explored the relation between characteristics of the sample and all congenital anomalies, metformin exposure, genetic syndromes, and non-genetic congenital heart defects by using the Pearson χ^2^ test for birth type and multiple birth and the χ^2^ test for trend for maternal age and gestational age at delivery. We calculated the risk of all non-genetic congenital anomaly (compared with genetic syndrome controls) in relation to pregestational/gestational diabetes, polycystic ovary syndrome, and infertility (all indications for metformin use) to assess the degree to which confounding by indication might be expected. We used logistic regression with listwise deletion to calculate odds ratios and 95% confidence intervals, adjusted for the confounders maternal age (<20, 20-24, 25-29, 30-34, 35-39, ≥40) and registry.

#### Analysis of risk of congenital anomaly associated with metformin exposure

We calculated odds ratios for the risk of each case congenital anomaly group/subgroup related to metformin exposure without adjustment and with adjustment for the confounding factors maternal age, registry, multiple birth (singleton, multiple birth), and maternal pregestational/gestational diabetes (yes/no). For stability of parameter estimates, we show results for only those subgroups with at least three cases exposed to metformin. Where the same number of exposed cases was observed in a congenital anomaly subgroup and a subgroup in a hierarchical level above, we analysed it at the lowest hierarchical level only. We used Stata version 12.1 for all analyses.

### Patient involvement

No patients were involved in setting the research question or the outcome measures, nor were they involved in developing plans for design or implementation of the study. No patients were asked to advise on interpretation or writing up of results. There are no plans to disseminate the results of the research to study participants or the relevant patient community. Affected families are thanked in the acknowledgments.

## Results

### Metformin use in study population and risk of congenital anomaly associated with disease indications

We recorded 53 689 babies affected by congenital anomaly in the EUROmediCAT database (2006-13), out of 1 892 482 births surveyed, across the 11 registries that were eligible to take part in this study. After exclusions (fig 1), 50 167 babies with congenital anomaly were left for analysis, consisting of 41 242 with a non-genetic anomaly and 8925 with a genetic syndrome.

In all, 168 babies affected by congenital anomaly (141 non-genetic and 27 genetic) were exposed to metformin (3.3 per 1000 babies affected by congenital anomaly), of which two had a combined preparation (A10BD02 metformin and sulfonylureas). The prevalence of metformin exposure varied across registries from 0.8 exposures per 1000 babies affected by congenital anomaly in Tuscany to 17.9 exposures per 1000 babies affected by congenital anomaly in Malta (table 1).

**Table 1:** Characteristics among all congenital anomaly affected babies, those exposed to metformin, and those with genetic syndromes and non-genetic congenital heart defects. Values are numbers (percentages) unless stated otherwise

	Babies with congenital anomaly	Metformin exposed	P value	Genetic syndromes	P value	Congenital heart defects as proportion of babies with non-genetic congenital anomalies	P value*
Total	50 167 (100)	168 (0.3)		8925 (17.8)		14 316/41 242 (34.7)	
**Registry (years)**							
Odense (2006-12)	1043 (2.1)	8 (0.8)		247 (23.7)		287/796 (36.1)	
Paris (2006-13)	6723 (13.4)	9 (0.1)		1827 (27.2)		1492/4896 (30.5)	
Tuscany (2006-13)	4980 (9.9)	4 (0.1)		1013 (20.3)		1537/3967 (38.7)	
Northern Netherlands (2006-13)	3410 (6.8)	6 (0.2)		757 (22.2)		846/2653 (31.9)	
Emilia Romagna (2006-13)†	5910 (11.8)	6 (0.1)		614 (10.4)		1865/5296 (35.2)	
Vaud (2006-13)	2288 (4.6)	3 (0.1)		558 (24.4)		725/1730 (41.9)	
Malta (2006-13)	780 (1.6)	14 (1.8)		110 (14.1)		265/670 (39.6)	
Saxony Anhalt (2006-13)	4237 (8.5)	6 (0.1)		511 (12.1)		1618/3726 (43.4)	
Wales (2006-13)	9286 (18.5)	59 (0.6)		1581 (17.0)		2411/7705 (31.7)	
Norway (2006-10)	8210 (16.4)	43 (0.5)		1807 (13.2)		2556/7129 (35.9)	
Reunion (2006-13)	3300 (6.6)	10 (0.3)		626 (19.0)		684/2674 (25.6)	
Missing	0	0		0		0	
**Maternal age (years)**							
<20	1764 (3.5)	0 (0.0)	0.02‡	170 (9.6)	<0.001‡	465/1594 (29.2)	<0.001‡
20-24	6920 (13.8)	17 (0.3)		741 (10.7)		2052/6179 (33.2)	
25-29	12 184 (24.3)	41 (0.3)		1391 (11.4)		3738/10 793 (34.6)	
30-34	14 766 (29.4)	57 (0.4)		2174 (14.7)		4453/12 592 (35.4)	
35-39	10 265 (20.5)	39 (0.4)		2652 (25.8)		2686/7613 (35.3)	
≥40	3742 (7.5)	14 (0.4)		1749 (46.7)		708/1993 (35.5)	
Missing	526 (1.1)	0 (0.0)		48 (9.13)		214/478 (45)	
**Multiple birth**							
Singleton	47 734 (95.2)	150 (0.3)	<0.001§	8636 (18.1)	<0.001§	13 437/39 098 (34.4)	<0.001§
Multiple birth	2202 (4.4)	18 (0.8)		253 (11.5)		822/1949 (42.2)	
Missing	231 (0.5)	0		36 (16)		57/195 (29)	
**Birth type**							
Live birth	41 140 (82.0)	138 (0.3)	0.09§	4136 (10.1)	<0.001§	13 398/37 004 (36.2)	<0.001§
Stillbirth	784 (1.6)	6 (0.8)		236 (30.1)		163/548 (29.74)	
Termination of pregnancy for fetal anomaly	8243 (16.4)	24 (0.3)		4553 (55.2)		755/3690 (20.46)	
Missing	0	0		0		0	
**Gestational length (weeks) (excluding terminations of pregnancy for fetal anomaly)**							
<28	830 (2.0)	4 (0.5)	0.003‡	138 (16.6)	<0.001‡	254/692 (36.7)	<0.001‡
28-31	1226 (2.9)	12 (1.0)		144 (11.8)		459/1082 (42.4)	
32-36	5663 (13.5)	25 (0.4)		840 (14.8)		1810/4823 (37.5)	
37-41	32 662 (77.9)	98 (0.3)		3090 (9.5)		10 524/29 572 (35.6)	
≥42	938 (2.2)	5 (0.5)		81 (8.6)		300/857 (35.0)	
Missing	605 (1.4)	0 (0.0)		79 (13.1)		214/526 (40.68)	

* Based on ratio of babies with non-genetic congenital heart defects to babies with other non-genetic congenital anomalies.

^†^ Excludes terminations of pregnancy for fetal anomaly.

‡ χ^2^ test for trend.

§ Pearson χ^2^ test.

From the exposure rate of 3.3 per 1000 and the total population surveyed of 1 892 482 births, we can estimated that approximately 6245 pregnancies in the surveyed population were exposed (assuming no or a small overall increased rate of metformin exposure among babies with congenital anomalies).

Metformin exposure was more common among older mothers, multiple pregnancies, and preterm births (table 1). The proportion of genetic syndromes, among all babies with congenital anomalies, was higher in older mothers, singleton births, terminations of pregnancy for fetal anomaly, and preterm births (table 1). Congenital heart defects, the largest group of congenital anomalies and one associated with diabetes, formed a higher proportion of all non-genetic congenital anomalies with increasing maternal age, in multiple births, and in preterm births and a lower proportion of terminations of pregnancy for fetal anomaly (table 1).

After exclusion of cases from Norway, where information on maternal illness was limited, 54.4% of mothers had either pregestational or gestational diabetes, including 44.0% with pregestational diabetes (table 2). Twenty four per cent had polycystic ovary syndrome, and 16.0% had infertility, indicated by an ICD-10 code for infertility or infertility treatment or exposure to a gonadotrophin or other ovulation stimulant. This left 18% (23/125) with no information available relating to the potential indication for metformin use (table 2).

**Table 2:** Number and percentage of metformin exposures with maternal diabetes, polycystic ovary syndrome (PCOS), and infertility or a combination of these diagnoses

Indication for metformin use	Metformin exposures
**All registries**	**(n=168)**
All diabetes (pregestational or gestational)	86 (51)
Pregestational diabetes	64 (38)
**Excluding Norway** *	**(n=125)**
Single indication:	
Pregestational or gestational diabetes	59† (47)
PCOS	16 (13)
Infertility	11 (9)
Two indications:	
Pregestational or gestational diabetes and PCOS	7‡ (6)
Pregestational or gestational diabetes and infertility	2‡ (2)
PCOS and infertility	7 (6)
No indication	23 (18)

* Owing to limited data on maternal illness from this registry.

† 51 (41%) with pregestational diabetes.

‡ 2 (2%) with pregestational diabetes.

The risk of all non-genetic anomalies compared with genetic controls was increased for maternal diabetes (pregestational or gestational) (adjusted odds ratio 2.04, 95% confidence interval 1.75 to 2.38) and maternal pregestational diabetes (2.51, 1.89 to 3.34). We found no evidence for an increased risk of all non-genetic anomalies compared with genetic controls for infertility (adjusted odds ratio 0.89, 0.66 to 1.19) or maternal polycystic ovary syndrome (0.81, 0.52 to 1.27).

### Analysis of risk of congenital anomaly with metformin exposure

We found no evidence for an increased risk of all non-genetic anomalies combined after exposure to metformin in the first trimester (adjusted odds ratio 0.84, 0.55 to 1.30) (table 3). Twenty nine subgroups of congenital anomaly had three or more metformin exposed cases (table 3). Among the anomalies not previously associated with pregestational diabetes in the EUROCAT database, the risk of ano-rectal atresia and stenosis was increased, compared with non-genetic and genetic controls. This association attenuated slightly on adjustment for confounders and was no longer statistically significant. Among the anomalies previously associated with pregestational diabetes, we found increased risk of atrial septal defect, pulmonary valve atresia, and “patent ductus arteriosus as the only congenital heart defect in liveborn term infants” compared with genetic and non-genetic controls. The atrial septal defect and patent ductus arteriosus associations attenuated on adjustment, with maternal diabetes being the main confounder (data not shown). The only signal to remain after adjustment for diabetes and other confounders was for pulmonary valve atresia, compared with non-genetic controls (table 3). Of the three metformin exposed cases with pulmonary valve atresia, one mother had maternal pregestational diabetes and the other two were exposed to ovulation stimulants, suggesting that metformin had been administered as part of infertility treatment.

**Table 3:** Number of congenital anomalies, number exposed, and odds ratios for metformin exposure in EUROCAT non-genetic congenital anomaly subgroups*

Case congenital anomaly subgroup	No (exposed)		Non-genetic controls (n=41 242; 141 exposed)				Genetic controls (n=8925; 27 exposed)	
			No† (exposed)	Odds ratio (95% CI)	Adjusted odds ratio^34^ (95% CI)		Odds ratio (95% CI)	Adjusted odds ratio‡ (95% CI)
All non-genetic congenital anomalies	41 242 (141)		-	-	-		1.13 (0.75 to 1.71)	0.84 (0.55 to 1.30)
**Anomalies not previously associated with pregestational diabetes in EUROCAT** 48								
Nervous system	4198 (14)		37 044 (127)	0.97 (0.56 to 1.69)	1.22 (0.70 to 2.15)		1.10 (0.58 to 2.11)	0.96 (0.49 to 1.92)
Oro-facial clefts	2623 (7)		38 619 (134)	0.77 (0.36 to 1.64)	0.75 (0.35 to 1.62)		0.88 (0.38 to 2.03)	0.74 (0.31 to 1.78)
Cleft lip with or without cleft palate	1601 (4)		38 619 (134)	0.72 (0.27 to 1.95)	0.73 (0.27 to 2.01)		0.83 (0.29 to 2.36)	0.78 (0.26 to 2.32)
Cleft palate	1022 (3)		38 619 (134)	0.85 (0.27 to 2.66)	0.78 (0.24 to 2.47)		0.97 (0.29 to 3.20)	0.73 (0.21 to 2.51)
Digestive system	3068 (15)		38 174 (126)	1.48 (0.87 to 2.54)	1.52 (0.88 to 2.63)		1.62 (0.86 to 3.05)	1.33 (0.67 to 2.63)
Ano-rectal atresia and stenosis	578 (5)		38 174 (126)	2.63 (1.07 to 6.47)	2.49 (0.98 to 6.29)		2.88 (1.10 to 7.50)	2.24 (0.80 to 6.30)
Diaphragmatic hernia	480 (4)		38 174 (126)	2.54 (0.93 to 6.89)	2.19 (0.78 to 6.14)		2.77 (0.97 to 7.95)	1.72 (0.56 to 5.29)
Urinary	6257 (23)		34 985 (118)	1.09 (0.70 to 1.71)	1.40 (0.89 to 2.22)		1.22 (0.80 to 2.12)	1.23 (0.68 to 2.22)
Multicystic renal dysplasia	795 (5)		34 985 (118)	1.87 (0.76 to 4.59)	1.91 (0.76 to 4.83		2.09 (0.80 to 5.43)	1.80 (0.66 to 4.92)
Congenital hydronephrosis	2217 (9)		34 985 (118)	1.20 (0.61 to 2.38)	1.33 (0.66 to 2.67)		1.34 (0.63 to 2.86)	1.23 (0.55 to 2.73)
Hypospadias§	3744 (8)		20 061 (65)	0.66 (0.32 to 1.37)	0.65 (0.31 to 1.37)		1.08 (0.40 to 2.87)	1.12 (0.38 to 3.32)
Limb	6716 (26)		34 526 (115)	1.16 (0.76 to 1.78)	1.07 (0.69 to 1.65)		1.28 (0.75 to 2.20)	0.91 (0.50 to 1.94)
Limb reduction	960 (5)		34 526 (115)	1.57 (0.64 to 3.84)	1.84 (0.74 to 4.62)		1.73 (0.66 to 4.49)	1.77 (0.65 to 4.81)
Clubfoot—talipes equinovarus	2238 (8)		34 525 (115)	1.07 (0.52 to 2.20)	0.91 (0.44 to 1.88)		1.18 (0.54 to 2.61)	0.83 (0.36 to 1.94)
Polydactyly	1824 (4)		34 526 (115)	0.66 (0.24 to 1.78)	0.69 (0.25 to 1.90)		0.72 (0.25 to 2.07)	0.64 (0.21 to 1.93)
**Anomalies previously associated with pregestational diabetes in EUROCAT** 48								
Neural tube defects	1587 (6)		37 044 (127)	1.10 (0.49 to 2.51)	1.36 (0.59 to 3.15)		1.25 (0.52 to 3.03)	1.12 (0.44 to 2.85)
Anencephalus and similar	565 (3)		37 044 (127)	1.55 (0.49 to 4.89)	2.15 (0.66 to 6.98)		1.76 (0.53 to 5.82)	1.66 (0.47 to 5.87)
Hydrocephalus	963 (3)		37 044 (127)	0.91 (0.29 to 2.86)	1.10 (0.34 to 3.53)		1.03 (0.31 to 3.40)	0.94 (0.27 to 3.24)
Congenital heart defects	14 316 (55)		26 926 (86)	1.20 (0.86 to 1.69)	0.95 (0.67 to 1.34)		1.27 (0.80 to 2.02)	0.83 (0.50 to 1.37)
Severe congenital heart defects	3600 (16)		26 926 (86)	1.39 (0.82 to 2.38)	1.05 (0.61 to 1.81)		1.47 (0.79 to 2.73)	0.94 (0.48 to 1.85)
Transposition of the great vessels	641 (4)		26 926 (86)	1.96 (0.72 to 5.36)	1.40 (0.50 to 3.93)		2.07 (0.72 to 5.93)	1.62 (0.53 to 4.95)
Ventricular septal defect	7338 (25)		26 926 (86)	1.07 (0.68 to 1.67)	0.94 (0.60 to 1.48)		1.13 (0.65 to 1.94)	0.81 (0.45 to 1.48)
Atrial septal defect	2840 (17)		26 926 (86)	1.88 (1.12 to 3.17)	1.47 (0.85 to 2.53)		1.98 (1.08 to 3.65)	1.45 (0.72 to 2.91)
Tetralogy of Fallot	532 (4)		26 926 (86)	2.36 (0.86 to 6.47)	2.03 (0.72 to 5.74)		2.50 (0.87 to 7.16)	2.16 (0.72 to 6.47)
Pulmonary valve stenosis	844 (4)		26 926 (86)	1.49 (0.54 to 4.06)	1.12 (0.40 to 3.14)		1.57 (0.55 to 4.50)	0.98 (0.32 to 2.95)
Pulmonary valve atresia	229 (3)		26 926 (86)	4.14 (1.30 to 13.20)	3.54 (1.05 to 12.00)¶		4.37 (1.32 to 14.53)	2.86 (0.79 to 10.30)
Patent ductus arteriosus as only congenital heart defect in liveborn term infants**	744 (6)		26 926 (86)	2.54 (1.11 to 5.82)	1.44 (0.60 to 3.43)		2.68 (1.10 to 6.51)	2.16 (0.77 to 6.03)
Omphalocele	350 (3)		40 892 (138)	2.52 (0.80 to 7.95)	2.83 (0.86 to 9.30)		2.85 (0.86 to 9.44)	2.41 (0.69 to 8.36)
Syndactyly	810 (3)		34 526 (115)	1.11 (0.35 to 3.51)	1.11 (0.34 to 3.55)		1.23 (0.37 to 4.05)	1.09 (0.31 to 3.80)

* Congenital anomaly subgroups with <3 metformin exposed cases were: encephalocele (1), spina bifida (2), microcephaly (1), eye (2), anophthalmos/microphthalmos (1), anophthalmos (1), ear, face, and neck (1), anotia (1), common arterial truncus (1), single ventricle (1), atrioventricular septal defect (1), aortic valve atresia/stenosis (1), hypoplastic left heart (1), coarctation of aorta (1), total anomalous pulmonary venous return (1), bilateral renal agenesis including Potter syndrome (1), posterior urethral valve and/or prune belly (2), craniosynostosis (2), congenital constriction bands/amniotic band (1), and situs inversus (2).

† No of controls used in each analysis will vary according to exclusion of case group and congenital anomaly subgroup at hierarchical level above where relevant.

‡ Adjusted for maternal age, registry, multiple birth, and maternal pregestational/gestational diabetes.

§ Male controls only.

¶ P=0.04.

** Gestational age ≥37 weeks.

## Discussion

We found no evidence of an overall increased risk of all major congenital anomalies combined after exposure to metformin during the first trimester.****Given the rise in exposure to metformin during pregnancy,^51^ ^52^ our findings are particularly timely. Our large international, population based database, with 168 cases of congenital anomaly exposed to metformin, from an estimated 6245 exposed pregnancies in Europe, represents more than five times the number of metformin exposures previously available in the literature.^22^ ^23^ ^24^ ^26^ Exposure to metformin during the study period remained rare; just over three in every 1000 babies affected by congenital anomaly were exposed to metformin in the first trimester, with considerable variation in the prevalence of metformin use across registries. Similar variation was evident across Europe in a study that used prescription redemption records.^52^ The variation in prescription of metformin between regions could be due to several factors—differences in the prevalence of pregestational diabetes, polycystic ovary syndrome, and infertility^53^ ^54^ ^55^; the diagnostic criteria used for polycystic ovary syndrome^56^; and the criteria for prescription of metformin for the different indications. The current prevalence of exposure to metformin during pregnancy would be expected to be higher because of the increasing prevalence of type 2 diabetes.^4^ ^5^ In keeping with the shared pathophysiological basis of the conditions for which metformin is indicated,^57^ some women had more than one indication for its use, also evidenced elsewhere.^51^

Our results are reassuring regarding the risk of all non-genetic congenital anomalies combined and support the previously available evidence.^22^ ^23^ ^24^ ^26^ Teratogens, however, tend to increase the risk of specific, rather than all, congenital anomalies,^58^ so focusing on specific congenital anomalies is important. We found a signal for pulmonary valve atresia. Pulmonary valve atresia has previously been associated with maternal diabetes,^48^ and our signal may suggest some residual confounding by indication. The number of comparisons made mean that this signal may also have arisen by chance. Pulmonary valve atresia has not been previously described after exposure to metformin during pregnancy.^22^ ^23^ ^26^ A recent teratogen information system cohort study found an elevated risk of cardiac defects following metformin exposure, but this was not significant and attenuated after adjustment for confounders.^26^

Although our findings are reassuring regarding the risk of congenital anomaly, further surveillance is recommended to increase the sample size and to follow up the pulmonary valve atresia signal in an independent dataset. The long term outcomes among babies who have been exposed to metformin in utero are also of interest. Metformin may have a direct influence on insulin action in the developing fetus, resulting in improved insulin sensitivity and a metabolically healthier pattern of growth into adulthood.^59^ ^60^

### Strengths and limitations of study

The main strength of this study is the use of the large international, population based, EUROmediCAT central database, the diverse nature of which improves the generalisability of our findings. EUROmediCAT also contains detailed and standardised coding of all congenital anomalies among live births and stillbirths, as well as terminations of pregnancy for fetal anomaly.^30^

Drug exposure in the EUROmediCAT database is mostly recorded prospectively, before anomaly status is known, which reduces the risk of recall bias. The exact timing of exposure within the first trimester is not recorded. It is likely that many metformin exposures would have been early in the first trimester during the critical period of development for many congenital anomalies, as women taking metformin for polycystic ovary syndrome or infertility are likely to stop their drug treatment when they realise they are pregnant. In most registries, normal clinical practice during our study period would have been to switch women who became pregnant while taking metformin for type 2 diabetes to insulin.

Drug data were not available for terminations of pregnancy for fetal anomaly from the Emilia Romagna registry. In Norway, drug exposure data were based on a prescription database, and we cannot be certain that mothers took the drug they collected at the pharmacy. Under-ascertainment of drug exposure in the EUROmediCAT database is known to occur for diseases other than diabetes and epilepsy, for which drugs are well recorded in medical records.^31^ ^61^ Comparison with Charlton et al suggests that metformin use may have been underreported in two of the registries.^52^ Any under-ascertainment of metformin exposure will be the same for cases and controls. This will have reduced the power of our analysis and may have slightly attenuated the estimated odds ratios. Teratogen non-specificity bias, whereby the exposure in question is associated with both cases and controls, may have attenuated odds ratios.^39^ To negate this, we used a very varied non-genetic control group, as well as a genetic control group.

We adjusted for confounding by indication by adjusting for maternal diabetes, but controlling for glycaemia (HbA_1c_) would have been more effective if this had been available.^62^ Other indications (polycystic ovary syndrome, infertility) were not associated in our data with the risk of non-genetic anomalies overall, but they may have confounded associations relating to specific subgroups.

In a fifth of metformin exposures, no information was available to suggest the reason for metformin use. Even in a large US cohort of insured pregnant women, where the nature of health insurance data would be expected to ensure that the indication for prescribing a drug was recorded, no indication for prescribing of metformin was available in 16.4% of cases.^51^ Where gestational diabetes was the only recorded indication (6% of those exposed to metformin), this may have been a first trimester diagnosis of gestational diabetes due to undiagnosed pregestational diabetes, or missing information on obesity/polycystic ovary syndrome,^3^ as these women are at high risk of going on to develop gestational diabetes.^57^

Owing to multiple testing of many congenital anomaly subgroups, some positive associations are likely by chance alone. We did not find more significant associations than would be expected by chance.

### Conclusion

We found no evidence of an overall increased risk of all major congenital anomalies combined after exposure to metformin during the first trimester. A signal for an increased risk of pulmonary valve atresia may be a chance finding. Although further surveillance is needed to increase sample size and follow up the cardiac signal, these findings are reassuring given the increasing use of metformin in pregnancy.

**What is already known on this topic**

Metformin affects stem cell function and has been shown to cross the human placenta at term, exposing the fetus to concentrations approaching those in the maternal circulation
Limited evidence from three meta-analyses and a cohort study suggests that the rate of all major congenital anomalies combined is not significantly increased after exposure to metformin
As teratogens tend to increase the risk of specific, rather than all, congenital anomalies, an increased risk of specific congenital anomalies after first trimester metformin exposure cannot be ruled out

**What this study adds**

In a large international, population based database, no evidence was found of an overall increased risk of congenital anomalies after first trimester metformin exposure
A raised risk of one specific cardiac defect may be a chance finding
Further surveillance is needed to increase sample size and follow up the cardiac signal, but these results are reassuring given the increasing use of metformin in pregnancy

## Web Extra

Extra material supplied by the author

## Notes

Contributors: JEG and HD had the idea for the study. JEG did the statistical analysis and wrote the first draft of the paper. ML advised on the conduct and coordination of the study, as well as interpretation of the results. EG and HD advised on interpretation of the results. EG, MCA, MB, MG, NL, KK, MM, AJN, AP, HR, and AR provided and verified the data. All authors had full access to all of the data (including statistical reports and tables) in the study and can take responsibility for the integrity of the data and the accuracy of the data analysis. All authors commented on drafts and read and approved the final manuscript. The corresponding author attests that all listed authors meet authorship criteria and that no others meeting the criteria have been omitted. JEG is the guarantor.

Ethical approval: Ethical approval was provided by the University of Ulster Nursing Research Governance Filter Committee.

Data sharing: EUROmediCAT aims to encourage the use of its data and networks for pharmacovigilance and drug safety research. Data sharing can be requested as per the ENCePP Code of Conduct—Implementation Guidance for Sharing of Study Data; see http://www.encepp.eu/code_of_conduct/documents/ENCePPCoCAnnex4_ImplementationGuidanceonSharingofENCePPStudyData.pdf and http://euromedicat.eu/currentresearchanddata/howtoproposeorcommissionspecificstudies for more information.

Transparency statement: The lead author affirms that this manuscript is an honest, accurate, and transparent account of the study being reported; that no important aspects of the study have been omitted; and that any discrepancies from the study as planned (and, if relevant, registered) have been explained.
