# Supplementary material for: Metformin exposure in first trimester of pregnancy and risk of all or specific congenital anomalies: exploratory case-control study
Source: BMJ. 2018 Jun 25;361:k2477. doi: 10.1136/bmj.k2477 (PMC6016021; doi:10.1136/bmj.k2477)
Supplement: Supplementary file 1 — Supplementary table [file givj043144.ww1.pdf]

Supplementary Table Sources of information on medication use in pregnancy in the EUROmediCAT registries included in this study (adapted from Supplementary Table 1. Beta-blocker use in pregnancy and risk of specific congenital anomalies: a European case-malformed control study, 2017[1] which was based on EUROCAT Special Report: sources of information on medication use in pregnancy, 2014[2] and EUROmediCAT registries descriptions, 2017.[3])

**Country**	**Registry**	**Prospective or retrospective**	**Information on which pregnancy outcomes**	**Medical files**			**Registry based data collection methods**
				From maternal health care providers in relation to pregnancy[1]	From health care providers of the child[2]	From pharmacies / prescription data	Interview / questionnaire
Denmark	Odense	P	LB, FD, TOPFA	X	X	-	-
France	Paris	P	LB, FD, TOPFA	X	X	-	-
	Isle de la Reunion	P	LB, FD, TOPFA	X	X	-	-
Italy	Tuscany	R	LB, FD, TOPFA	-	-	-	X[3]
	Emilia Romagna	P	LB, FD	X	X	X[4]	-
The Netherlands	Northern Netherlands	P,R	LB, FD, TOPFA	X	X	X[5]	X[6]
Malta[7]	Malta	P	LB, FD	X	X	-	-
Germany	Saxony Anhalt	P	LB, FD, TOPFA	X	X	-	-
United Kingdom	Wales	P	LB, FD, TOPFA	X	X	-	-
Norway	Norway	P	LB, FD, TOPFA	-	-	X[8]	-

P, prospective, medication was recorded before outcome of pregnancy was known; R, retrospective
LB, live births; FD, foetal deaths ≥ 20 weeks of gestational age (including stillbirths); TOPFA, terminations of pregnancy for foetal anomaly
[1] includes midwife, obstetrician, gynaecologist, delivery units, General Practitioner, pregnancy pass
[2] includes paediatrician, neonatologist, geneticist, paediatric cardiologist, paediatric neurologist, paediatric surgeon
[3] interviews performed by gynaecologists, paediatricians, geneticists and other specialists using a standardised questionnaire
[4] Emilia Romagna Prescription Database, in which prescriptions are recorded irrespective of pregnancy status
[5] medicine delivery data from community pharmacies, which are recorded irrespective of pregnancy status

[6] maternal medication use of drugs (including the use of prescribed and 'over the counter' drugs, but also on folic acid, multivitamins, vaccinations, anaesthetics by dentists etc.) is a standard item in the questionnaire. If any answer about the actual drug use was unclear, it was clarified in an interview by the registry staff. The interview always took place after the birth and was carried out for all types of births.
[7] TOPFA is illegal in Malta
[8] prescription database, in which prescriptions are recorded irrespective of pregnancy status

References

1. Bergman JEH, Lutke LR, Gans ROB, et al. Beta-Blocker Use in Pregnancy and Risk of Specific Congenital Anomalies: A European Case-Malformed Control Study. Drug Saf [Internet]. 2017 Dec 11;1–13. Available from: https://doi.org/10.1007/s40264-017-0627-x
2. Bakker M, de Jonge L. EUROCAT Special Report: Sources of Information on Medication Use in Pregnancy [Internet]. Bakker, M; de Jonge, L; Newtownabbey: EUROCAT Central Registry; 2014. Available from: http://www.eurocat-network.eu/content/Special-Report-Medication-Use-In-Pregnancy.pdf
3. EUROmediCAT Central Database. EUROmediCAT Registry Descriptions [Internet]. EUROmediCAT Website. Belfast; 2017. p. 1–96. Available from: http://euromedicat.eu/content/Partners-Registry-Descriptions-February-2017.pdf
